# Supplementary material for: The causal role of circulating inflammatory markers in osteoporosis: a bidirectional Mendelian randomized study
Source: Front Immunol. 2024 Jul 18;15:1412298. doi: 10.3389/fimmu.2024.1412298 (PMC11291241; doi:10.3389/fimmu.2024.1412298)
Supplement: Supplementary file 1 [file DataSheet_1.zip › supplementary material/OSTEOPOROSIS-CIM/GCST90274779.tsv.gz_leave_one_out.pdf]

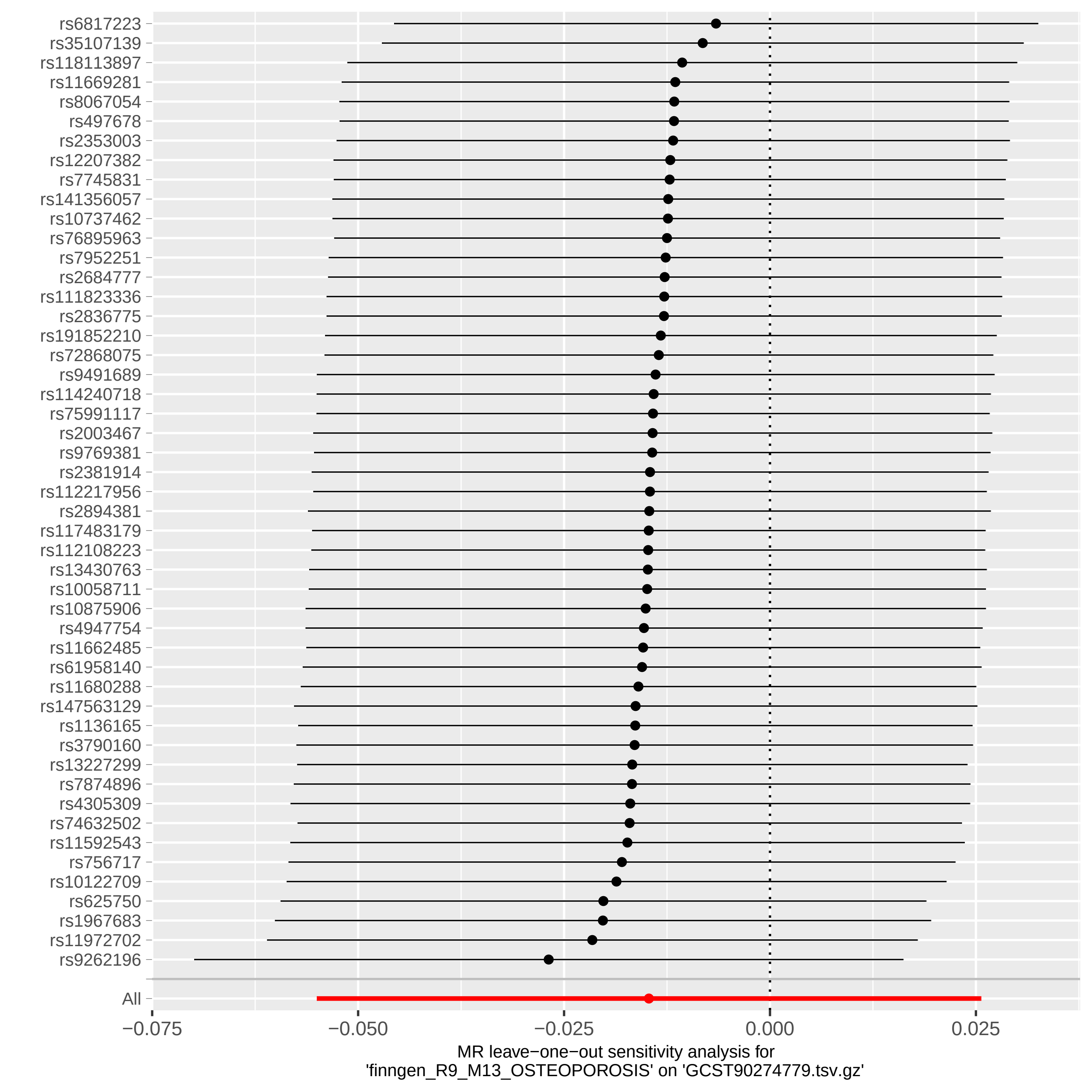
rs6817223
rs35107139
rs118113897
rs11669281
rs8067054
rs497678
rs2353003
rs12207382
rs7745831
rs141356057
rs10737462
rs76895963
rs7952251
rs2684777
rs111823336
rs2836775
rs191852210
rs72868075
rs9491689
rs114240718
rs75991117
rs2003467
rs9769381
rs2381914
rs112217956
rs2894381
rs117483179
rs112108223
rs13430763
rs10058711
rs10875906
rs4947754
rs11662485
rs61958140
rs11680288
rs147563129
rs1136165
rs3790160
rs13227299
rs7874896
rs4305309
rs74632502
rs11592543
rs756717
rs10122709
rs625750
rs1967683
rs11972702
rs9262196
All
−0.075
−0.050
−0.025
0.000
0.025
MR leave−one−out sensitivity analysis for
'finngen_R9_M13_OSTEOPOROSIS' on 'GCST90274779.tsv.gz'
